# Innate immunity to SARS-CoV-2 infection: a review

**DOI:** 10.1017/S095026882200125X

**Published:** 2022-07-18

**Authors:** Marcos Jessé Abrahão Silva, Yan Corrêa Rodrigues, Karla Valéria Batista Lima, Luana Nepomuceno Gondim Costa Lima

**Affiliations:** 1 Graduate Program in Epidemiology and Health Surveillance (PPGEVS) of the Evandro Chagas Institute (IEC), Ananindeua, Pará, Brazil; 2 Bacteriology and Mycology Section of the Evandro Chagas Institute (IEC), Ananindeua, Pará, Brazil

**Keywords:** COVID-19, cytokines, immunity, innate immunity, SARS-CoV-2

## Abstract

The severe acute respiratory syndrome coronavirus 2 (SARS-CoV-2) pandemic, first notified in China, has spread around the world causing high morbidity and mortality, which is due to factors such as the subversion of the immune response. The aims of the study are to summarise and present the immunopathological relationship of COVID-19 with innate immunity. This is a systematic review conducted by the National Library of Medicine – National Institutes of Health, USA (PUBMED), Latin American and Caribbean Literature on Health Sciences (LILACS), Medical Literature Analysis and Retrieval System Online (MEDLINE) and Scientific Electronic Library Online (SCIELO) databases with clinical trials, *in vitro* assays, case-controls, cohort studies, systematic reviews and meta-analyses between February 2020 and July 2021. The version 2 of the Cochrane risk-of-bias tool for RCTs (RoB 2), Joana Briggs Institute (JBI) Critical Appraisal (for the review articles) and the Risk of Bias in Non-randomised Studies of Interventions (ROBINS-I) tools were used to evaluate the quality and the risk of bias of the studies included in this review. The innate immune response through the generation of interferons, alternative pathways and complement system lectins and the joint action of innate immune cells and cytokines and chemokines lead to different clinical outcomes, taking into account the exacerbated inflammatory response and pathogenesis. Then, in addition to interacting as a bridge for adaptive immunity, the innate immune response plays an essential role in primary defense and is one of the starting points for immune evasion by SARS-CoV-2.

## Introduction

Living organisms have two key barriers against possible harmful invaders: the biological barrier, such as the skin, and the immune barrier. In humans, it is possible to distinguish the latter between innate and adaptive immunity. Innate immunity is the host's first line of defense against microbial infectious agents. During the antiviral response, the innate response is responsible for quickly detecting and defending against invaders, while creating a bridge to the adaptive response [1].

Mammalian antigen presenting cells (APCs), such as macrophages and dendritic cells (DCs) are able to detect microbes through pattern recognition receptors (PRRs), most notably Toll-like receptors (TLRs), retinoic acid inducible gene I-like receptors (RIG-I), such as RIG-like receptors (RLRs), the nucleotide-binding oligomerisation domain (NOD)-like receptor family proteins (NLRs) and absent in melanoma 2 (AIM2). Through PRRs, such as mainly AIM2 and NLR in contact with pathogen-associated molecular patterns (PAMPs) and damage-associated molecular patterns are able to induce the formation of the inflammasome (a large multiprotein complex that is responsible for processing pro-inflammatory cytokines and creating membrane pores), which leads to a cell death called pyroptosis [2–4].

The innate immune system creates interferons (IFNs) and cytokines essential for elimination of viral agents. However, innate immune signalling must be well regulated, as over-activation can lead to systemic inflammation and tissue damage to the host [5].

Coronaviruses are a group of single-stranded, positive-sense viruses belonging to the family Coronaviridae and subfamily Orthocoronavirinae that are largely responsible for common colds in humans. The family Coronaviridae is subdivided into Torovirinae and Coronavirinae which contains the genera Alphacoronavirus, Betacoronavirus, Gammacoronavirus and Deltacoronavirus. However, in turn, in December 2019, there was the first record of coronavirus disease 2019 (COVID-19) in Wuhan, China. The aetiological agent is the severe acute respiratory syndrome coronavirus 2 (SARS-CoV-2), of the genus Betacoronavirus, of zoonotic origin has perpetuated itself around the world causing a pandemic announced in March 2020 by the World Health Organization (WHO), which is already reported to be the cause of more than 3 million deaths and more than 150 million ill. Infected senior citizens with comorbidities had the worst prognosis [6].

COVID-19 is a highly contagious severe respiratory disease. It has been shown to be more infectious than other coronavirus types that have created outbreaks in humans, such as SARS-CoV and Middle East respiratory syndrome (MERS-CoV), with several viral strategies for evading the host immune response. Some of them are involved in inhibitory devices of the innate immune response [7].

This family of viruses has in particular a long RNA strand and a peculiar replication strategy. SARS-CoV-2 has an RNA strand of about 29.9 kb in length. Like other coronaviruses, this virus has at least six extra open reading frames (ORFs) in its genome. The first ORFs (ORF1a/b) are about two-thirds of the entire genome length and encode 16 non-structural proteins closer to the 5′ end (nsps 1–16). These ORFs produce two polypeptides, including p1a and pp1ab. The other one-third of the genome corresponds to structural proteins already present near the 3′ region [8].

SARS-CoV-2 has four structural proteins, known as S (spike), E (envelope), M (membrane) and N (nucleocapsid) proteins and the genes for accessory proteins (protein (HE), 3, 7a, among others). The S protein binds to the human ACE2 receptors (angiotensin-converting enzyme 2, a transmembrane protein), transferring the genetic material to the cell interior, initiating the replication process. After binding to the receptor, the initiation of protein S is mediated by transmembrane serine protease 2 (TMPRSS2), a transmembrane serine protease of the host cell, which involves cleavage of protein S at the sites S1/S2 and S2 [9].

Physiologically, the typical RAS pathway consists of a series of steps that ultimately catalyse the formation of angiotensin (Ang) II, which promotes vasoconstriction, sodium reabsorption and water retention to elevate blood pressure. Ang II causes these effects by binding to and activating the Ang II type 1 receptor (AT 1R). Subsequently, the ACE2 axis, an anti-regulatory arm of the RAS, was detected. ACE2 inactivates Ang II by converting it to Ang 1–7, a peptide with anti-inflammatory, antifibrotic and vasodilatory properties thanks to activation of the Mas receptor (MasR). Therefore, ACE2 can act as a receptor for SARS-CoV-2, but has a protective effect on the RAS pathway [10].

In most patients, the recruited cells destroy the pathogen and then the immune response is reduced in a controlled manner. However, in some cases, this cellular recruitment brings about lung infiltration by immune system cells, triggering a hyperinflammatory response caused by exacerbated cytokines, which is a determinant grievance for patients with comorbidities, since as already mentioned it can cause tissue damage and even systemic, being a predictive factor for death [11].

In this context, the following research problem arises: ‘What immunopathological aspects of SARS-CoV-2 in innate immunity are involved in the progression of COVID-19?’ The aim of this paper is to answer the problem and to build a didactic model of the innate immune response in COVID-19.

## Methods

### Study design

This is a systematic review to collect concise, complete and recent data on innate immunity in COVID-19. The PRISMA flowchart tool that is part of the Preferred Reporting Items for Systematic Review and Meta-Analysis Protocols (PRISMA protocol) was used to show how the final sample was arrived at, describing all the steps, inclusions and exclusions.

The study followed the following training steps: (1) preparation of the guiding question; (2) stipulation of inclusion and exclusion criteria; (3) choice of articles; (4) analysis of articles (5) interpretation, discussion and presentation of the review [12]. To create the guiding question the PICO strategy was used, related to the following categories: Population; Intervention; Comparison; Outcome [13]. In this way, the question is described by: Population, are patients with COVID-19; Intervention is about evaluating the immunopathogenic aspects in patients with SARS-CoV-2 infection; Comparison is to relate the immunopathogenic aspects of the virus and innate immunity; Outcome resides in disease progression. Then, it resulted in: ‘What immunopathogenic aspects of SARS-CoV-2 in innate immunity are involved in the progression of COVID-19?’

### Data sources

The search terms that were used for the search based on the Medical Subject Headings (MeSH) are: ‘SARS-CoV-2’; ‘COVID-19’; ‘Innate Immunity’; ‘Immunity’; ‘Cytokines’. Articles were searched with the combination of the descriptors and Boolean operator ‘AND’. The following databases were used in the literature search: National Library of Medicine National Institutes of Health of the USA (PUBMED), Latin American and Caribbean Literature in Health Sciences (LILACS), Medical Literature Analysis and Retrieval System Online (MEDLINE) and Scientific Electronic Library Online (SCIELO).

### Study eligibility criteria and participants

Articles published from February 2020 to July 2021, available complete, original, narrative reviews, systematic reviews, clinical trials, *in vitro* assays, quasi-experiments, ecological studies, comparative studies, cross-sectional studies, case series, case-controls, cohort studies (prospective and retrospective) and meta-analyses were defined as inclusion criteria, and could be in English or Spanish. The exclusion criteria were articles published before 2020 and after July 2021, articles with only abstracts available, letters to the editor and articles or materials with topics not pertinent to the research question. The participants of the study were people infected by the SARS-CoV-2 infection.

### Quality assessment and risk of bias

Two authors (MJAS and YCR) independently used the version 2 of the Cochrane risk-of-bias tool for RCTs (RoB 2), Joana Briggs Institute (JBI) Critical Appraisal (for the review articles) and the Risk of Bias in Non-randomised Studies of Interventions (ROBINS-I) tools for evaluating the methodological quality and the risk of bias of the studies [14–16]. Any discrepancies during the process were ironed out with the help of a third author (KVBL).

### Data collection and extraction

Two researchers (MJAS and YCR) independently extracted data from the included publications in accordance with a predefined procedure. The same two researchers (MJAS and YCR) also assessed and organised the data in Microsoft Office Excel 365, collecting the following information: (1) title; (2) database; (3) methodology; (4) results relevant to the research topic. These observations have been represented below in a tabular form. Any discrepancies were ironed out with the help of a third author (KVBL).

## Results

**Fig. 1.**
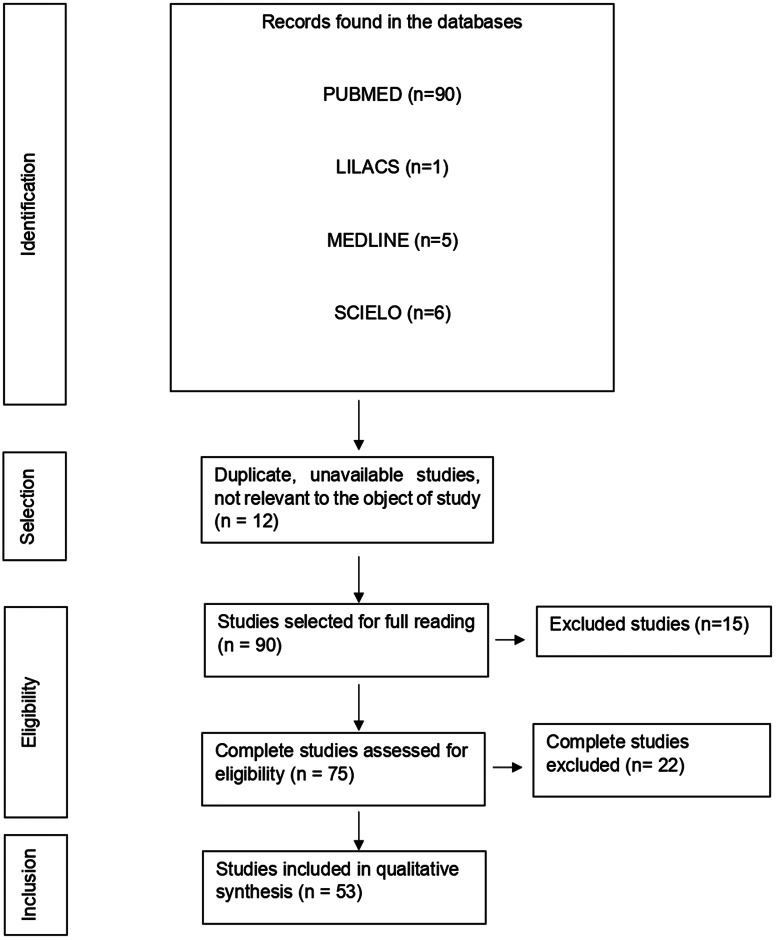
Flowchart of procedures for identification, selection, eligibility and inclusion of studies for analysis. Belém, PA, Brazil (2021).

**Fig. 2.**
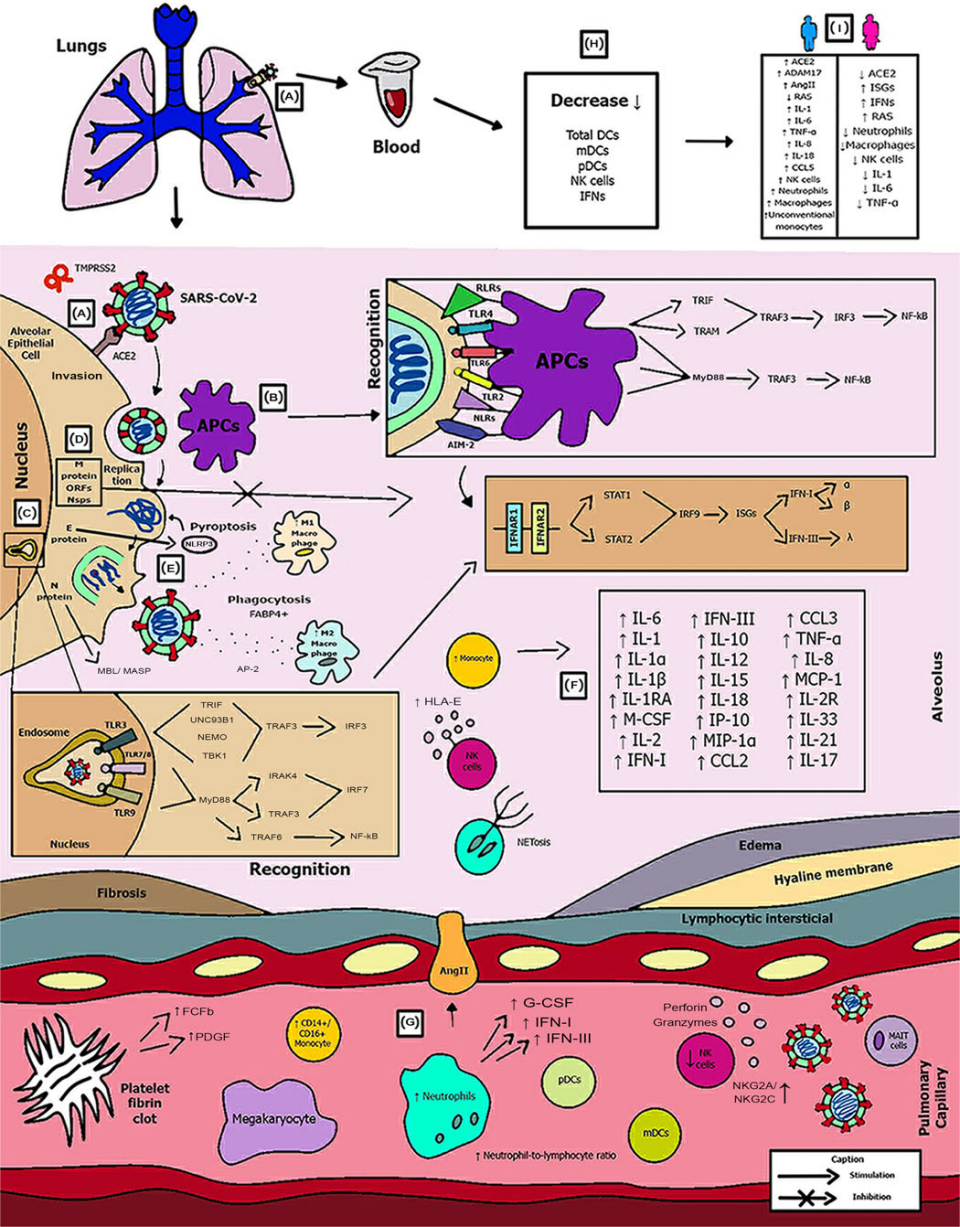
Schematic model of innate immunity in SARS-CoV-2 infection.

With the application of the inclusion criteria, a total of 102 articles were obtained; however, some articles were letters to the editor, they were not available in full or provided information that was not relevant to the research question (Fig. 1). Thus, the final framework of articles consisted of 53 articles (Table 1). The articles were entirely international (53) derived from PUBMED, LILACS, MEDLINE and SCIELO. The didactic model was constructed based on the results achieved by the bibliographic search in Table 1 (Fig. 2).

**Table 1.** Characteristics of the studies included in the systematic review

No.	Database	Methodology	Results	Reference
1	PUBMED	*In vitro* assay	SARS-CoV-2 was shown to be sensitive to IFN-α and IFN-β treatment in cell culture.	[17]
2	PUBMED	*In vitro* assay	Human intestinal epithelial cells (hIECs) supported the infection, replication and production of SARS-CoV-2 viral particles, contributing to increased viraemia and generating in the patient the enteric phase of SARS-CoV-2 and an exacerbated cytokine response. Viral infection was found to provoke an IFN-III-mediated immune response, which is efficient in controlling SARS-CoV-2 replication.	[18]
3	PUBMED	Clinical trial	The NSP1, NSP3, NSP12, NSP13, NSP14, ORF3, ORF6 and M proteins of SARS-CoV-2 inhibit Sendai virus-induced IFN-β promoter activation, whereas NSP2 and S proteins exert opposite effects. Further analysis indicates that ORF6 inhibits type I interferon and downstream signalling, and that C-terminal region of this protein is crucial for its antagonistic effect.	[19]
4	PUBMED	Clinical trial	ORF6, viral ORF8 and the nucleocapsid (N) proteins were potential inhibitors of the type I interferon signalling pathway.	[20]
5	PUBMED	Review	It presents results that macrophages, neutrophils, mature dendritic cells (mDC), pDCs, NK (natural killer) cells, T cells, B cells, plasma cells and epithelial cells were found in all groups analysed, although in different proportions according to disease severity. It can be inferred that the substantial recruitment into the lung of pro-inflammatory immune cells, particularly macrophages and neutrophils, may contribute, especially in patients with COVID-19, to the occurrence of severe symptoms of excessive inflammation, resulting in systemic manifestations and multiple organ dysfunctions.	[21]
6	PUBMED	*In vitro* assay	There is strong activation of NK cells in distinct subsets in the peripheral blood of patients with COVID-19. The characteristics of these immunotypes were high expression of perforin, NKG2C (receptor for NK cells) and Ksp37 (37 kD specific killer secretory protein). SARS-CoV-2 RNA activates the RIG-I-MAVS-dependent IFN signalling pathway. The overall number of NK cells (CD56+) is substantially reduced in the blood of COVID-19 patients compared to healthy controls.	[22]
7	PUBMED	*In vitro* assay	SARS-CoV-2 RNA is shown to activate the RIG-I-MAVS (mitochondrial antiviral signalling protein) dependent IFN signalling pathway. Furthermore, ORF9b immediately accumulates and antagonises the IFN type I antiviral response during SARS-CoV-2 infection.	[23]
8	PUBMED	Clinical trial	A close association of decreased DCs and increased monocytes similar to myeloid-derived suppressor cells (MDSCs) was found, which correlated with lymphopaenia and inflammation in the blood of patients with severe COVID-19. In contrast, monocyte-macrophages in BALFs (in bronchoalveolar lavage fluid) from patients with COVID-19 produced large amounts of cytokines and chemokines but secreted few IFNs. Interestingly, relatively higher levels of anti-inflammatory cytokines (IL-1R2, IL-1RN and TGF (tumour growth factor)-B1) and lower levels of IL-18 were observed in BALF monocyte-macrophages from COVID-19 was severe than in mild cases, whereas classical proinflammatory cytokines (IL-1A, IL-1B, IL-6) and TNF were comparable between the two groups. Monocyte and neutrophil recruitment chemokines (CCL2 (CCL – chemokine ligand), CCL3, CCL4, CCL7, CCL8, CXCL1 (CXC chemokine ligand 1), CXCL2, CXCL3 and CXCL8) were highly expressed, while CL9 and chemokines (CXCL16) recruiting T cells were less expressed by monocyte-macrophages in BALFs from severe COVID-19 than those in mild cases.	[24]
9	PUBMED	Review	It was observed that SARS-CoV-2 activates alveolar, splenic and renal macrophages through ACE2 and increases the secretion of IL-6, TNF-α and IL-10. Furthermore, macrophages can contribute to tissue regeneration and return to homoeostasis.	[25]
10	PUBMED	Clinical trial	There were no significant differences in the number of monocytes between patients with COVID-19 and normal healthy individuals. However, more pronounced significant morphological and functional differences were identified in patients with prolonged hospitalisation and admission to the intensive care unit (ICU). Flow cytometry provided significant results regarding the characterisation of monocytes in COVID-19.	[26]
11	PUBMED	*In vitro* assay	SARS-CoV-2 ORF3b is a potent IFN antagonist, suppressing type I IFN induction more efficiently than SARS-CoV. Phylogenetic analyses and functional assays revealed that SARS-CoV-2-related viruses from bats and pangolins also encoded truncated ORF3b gene products with strong anti-IFN activity.	[27]
12	PUBMED	*In vitro* assay	It has been shown that nsp13, nsp14, nsp15 and orf6 of SARS-CoV-2, but not the only orf8, can potentially suppress primary IFN signalling and production. orf6 demonstrated the greatest suppression in primary IFN signaling and production and sin. The PLpro enzyme, despite sharing high amino acid sequence similarity with SARS-CoV, loses IFN and deubiquitinase antagonising activities.	[28]
13	PUBMED	*In vitro* assay	The host's innate immune response has been shown to be driven by multiple viral proteins, among which ORF6 potently disrupts signalling pathways both upstream and downstream of IFN production. NSP1, NSP3, NSP13, NSP14, ORF6, ORF8, N and S inhibited IFN-β induced ISRE promoter activity, while NSP6, NSP9, NSP12 and E proteins showed stimulatory effects. In addition, SARS-CoV-2 has been shown to be susceptible to IFN treatment. ORF6 inhibits IFN signalling.	[19]
14	PUBMED	Case-control	Compared to healthy controls, patients infected with SARS-CoV-2 express inflammatory cytokines and chemokines such as IL-1β, IL-1 receptor antagonist (IL1RA), IL-2, IL-6, IL-7, IL-10, TNF-α, IFN-γ, high plasma concentrations of macrophage colony stimulating factors (M-CSF), granular cell stimulating factor (G-CSF), fibroblast growth factor (FCFb), platelet-derived growth factor (PDGF), vascular endothelial growth factor (VEGF), CCL2 chemokine ligand 2 (CCL2), CCL3, CCL chemokine ligand 10 (CXCL10), CCL8, CXCL2, CXCL8, CXCL9, CXCL16. In severe cases of COVID-19, serum levels of IL-1, IL-2, IL-6, IL-7, IL-10, TNF-α, G-CSF, CCL2, CCL3, CXCL8, CXCL10 were elevated.	[29]
15	PUBMED	Cohort study	The production of proinflammatory cytokines and chemokines induced by SARS-CoV-2 was observed in symptomatic and asymptomatic patients, returning to normal after recovery. IL-6, IL-7, IL-10, IL-18, G-CSF, M-CSF, MCP (monocyte chemoattractant protein)-1 (also known as CCL2), MCP-3 (monocyte chemotactic protein 3, also known as CCL7), IP (inducible protein)-10, MIG (IFN gamma-induced monokine, also known as CXCL9) and MIP-1α (macrophage 1-alpha inflammatory protein, also known as CCL3) were associated with severity of COVID-19. In addition, a set of cytokine and chemokine profiles (IL-1β, IL-2, IL-2Rα, IL-3, IL-4, IL-7, IL-8, IL-12 (p40), GM-CSF, IFN-α2, IFN-γ, basic FGF (fibroblast growth factor), NGF (nerve growth factor)-β, HGF (hepatocyte growth factor), LIF (leukaemia inhibitory factor), VEGF (leukaemia factor vascular endothelial growth), MCP-3 and MIP-1α) were significantly higher in male patients infected with SARS-CoV-2 than in female patients. Serum levels of MCP-1, G-CSF and VEGF were weak and positively correlated with viral titres.	[30]
16	PUBMED	Meta-analysis	Neutrophil counts as well as neutrophil-to-lymphocyte ratio (NLR) were increased. On the other hand, low lymphocyte-to-C-reactive protein ratios were detected in patients with severe COVID.	[31]
17	PUBMED	Review	Increased levels of TLR3 transcription after coronavirus infections are detected as early as day 2 post-infection; this determines the activation of downstream molecules such as TRIF (IFN-β inducing adaptor containing the TIR domain), which determines the activation of transcription factors such as IRF3 (interferon regulatory factor 3) and NF-κB, associated with increased production of type I interferons (IFN-alpha and beta), inflammatory cytokines (IL-6, TNF) and IFN-gamma. The production of oxidised phospholipids by viruses characterised by lung damage causes the activation of TLR4, similar to bacterial LPS (lipopolysaccharide), which determines the activation of MyD88 (myeloid differentiation factor 88) and TRIF, with consequent overproduction of inflammatory cytokines. TLR7 and TLR8 have shown implication in the coronavirus response. TLR7 recognises single-stranded RNA in endosomes, such as RNA from viruses like coronaviruses. TLR7 is highly expressed in human pDCs and B cells. TLR7 binds ssRNA and activates the MyD88 pathway, with consequent activation of the mitogen-activated protein kinase (MAPK) cascade, NF-κB and other pathways. This activation increases the expression of TNF-alpha, IL-1beta, IL-6, IL-12 and IFN-alpha. TLR8 is expressed in myeloid cells and low levels in pDCs and similar to TLR7 localises to endosomes. RNA degradation in endosomes causes its binding to TLR8 with consequent conformational change and activation of MyD88 with a descending pathway similar to TLR7. In various forms of coronavirus-mediated SARS, neutrophils and macrophages play a pathogenetic role in alveolar damage as their localisation correlates with the most damaged sites in lung tissue, activating complement system. In some individuals, this response appears to be aberrant, causing immune-mediated damage in patients even more damaging than the viral damage itself.	[32]
18	PUBMED	Cohort study	Severe cases tend to have lower lymphocyte counts, high white cell counts and high neutrophil-to-lymphocyte ratio (NLR), as well as lower rates of monocytes, eosinophils and basophils.	[33]
19	PUBMED	Cohort study	The rising neutrophil counts parallel the computed tomography (CT) scan values of the lesion, reflecting the neutrophil-induced lung injury in critically ill patients. Transcriptome analysis revealed that neutrophil activation was correlated with 17 genes associated with the extracellular neutrophil trap (NET) in COVID-19 patients, which was related to innate immunity.	[34]
20	PUBMED	Case-control	The genes encoding the inhibitory receptors LAG3 (lymphocyte activator gene 3) and TIM3 (T cell immunoglobulin and mucin-3 domain) are up-regulated in NK cells from COVID-19 patients. An impaired type I IFN response has been identified in critically ill COVID-19 patients, accompanied by elevated blood viral load and an excessive NF-κB-induced inflammatory response associated with increased TNF-α and IL-6. It was also observed that SARS-CoV-2 infection was characterised by an absence of circulating IFN-β in COVID-19 patients with all degrees of disease severity. Furthermore, the most severe patients with COVID-19 exhibited impaired IFN-α production that was associated with lower viral clearance.	[35]
21	PUBMED	Case-control	In patients with COVID-19 a negative correlation was found between serum IL-6 amounts and the absolute number of HLA-DR molecules in CD14 monocytes, and between the absolute lymphocyte count and the absolute number of mHLA-DR (human leucocyte antigen molecules) in CD14 monocytes of patients with COVID-19.	[36]
22	PUBMED	Case-control	A substantial reconfiguration of the peripheral immune cell phenotype in COVID-19, including a heterogeneous ISG signature, negative HLA class II regulation and a novel B-cell-derived granulocyte population in patients with acute respiratory failure requiring mechanical ventilation was identified. Also, peripheral monocytes and lymphocytes do not express substantial amounts of pro-inflammatory cytokines, suggesting that circulating leucocytes do not contribute significantly to the potential COVID-19 cytokine storm.	[37]
23	PUBMED	Cohort study	The proportion of patients with elevated IL-2R, IL-6, IL-8, IL-10 and TNF was higher among severe cases of the disease. The amounts of IL-2R, IL-6, IL-8, IL-10 and TNF were also two- to 20-fold higher among patients who went on to obit compared with those who recovered. In multivariate analysis, higher levels of IL-2R and IL-6 on admission were associated with increased likelihood of in-hospital death, independent of other variables, including disease severity and lymphocyte count.	[38]
24	PUBMED	Meta-analysis	At least 3.5% of patients with life-threatening COVID-19 pneumonia showed autosomal recessive deficiencies in IRF7 and IFNAR1 (alpha/beta-interferon receptor subunit 1) genes and autosomal-dominant deficiencies in genes encoding TLR3, Unc-93B1 homologue, TLR molecule adaptor 1, TBK1 (TANK-binding kinase 1, also known as T2K), IRF3, IRF7, IFNAR1 and IFNAR2.	[39]
25	PUBMED	*In vitro* assay	Type I interferon (IFN-I) immune activity was found to be attenuated in COVID-19 cases, contributing to the pathogenicity of SARS-CoV-2. The N protein of SARS-CoV-2 exhibited a significant inhibitory effect on IFN-2α- or IFN-β-induced activation of the ISRE promoter, indicating that the N protein may act as an antagonist of IFN signalling. Ectopic expression of N from SARS-CoV-2 did not affect the steady-state levels of STAT1 and STAT2, while phosphorylation of STAT1 and STAT2 (p-STAT1 in Tyr701 and p-STAT2 in Tyr690) was reduced in cells expressing N protein compared to those in control cells. N protein can interact with STAT1 and STAT2 and suppress their IFN-induced nuclear translocation. Viral RNA replication was significantly elevated in SARS-CoV-2-infected cells in the presence of N overexpression.	[40]
26	PUBMED	Clinical trial	Age group and obesity were factors associated with reduced and/or altered IFN-α and IFN-β responses. After day 10, IL-6 remains increased while IFN-α decreases.	[41]
27	PUBMED	Review	The association of aberrant NET formation and pulmonary manifestations, thrombosis, mucous airway secretions and cytokine production in patients with COVID-19 was shown.	[42]
28	PUBMED	Cohort study	Among the biomarkers analysed, eight of them (IL-15, IL-2, NGAL (neutrophil gelatinase-associated lipocalin), CCL2, MMP-9 (matrix metallopeptidase 9), sTNFRSF1A (soluble tumour necrosis factor receptor 1A superfamily), sST2 (soluble ST2, also known as interleukin 1 (IL1RL-1) and IL-33 receptor, IL-10) and two additional biomarkers (lactoferrin, CXCL9) were substantially associated with increased mortality, whereas IL-1α was associated with decreased mortality. Among these, sST2, sTNFRSF1A, IL-10 and IL-15 were consistently higher throughout hospitalisation in patients who died *vs.* those who recovered. Neutrophils exhibited extensive vacuolisation consistent with an activated state, and blood levels of four neutrophil-derived molecules (MMP-9, NGAL, lactoferrin and S100A9) showed a positive correlation with the risk of death. In this study, the levels of IL-1β, IL-1RA, IL-18 and IL-18BP were higher in patients with COVID-19 than in healthy individuals.	[43]
29	PUBMED	Clinical trial	Data show that SARS-CoV-2 infects mainly lung epithelial cells and macrophages, but only some macrophages and monocytes from BALFs have elevated levels that showed excess pro-inflammatory cytokines.	[44]
30	PUBMED	Cohort study	The NKG2A immune checkpoint is increased with a reduced ability to produce CD107a, IFN-γ, IL-2, granzyme B and TNF-α on NK cells and CD8+ T cells for early stage COVID-19 disease progression.	[45]
31	PUBMED	Case-control	In patients with COVID-19, positive regulation of a multitude of pro-inflammatory cytokines has been observed, suggesting the pathogenic role of hypercytokinaemia. The most prominent feature of the cytokine profile is elevated expression of multiple chemokines and their receptors. Among them, several neutrophil chemoattractants were positively regulated. Among innate immune cells, activated DCs, activated mast cells and neutrophils were more abundant in the groups with viral pneumonia compared to healthy ones. SARS-CoV-2 triggered a robust IFN response, marked by the expression of several ISGs, including interferon-induced transmembrane proteins (IFITMs).	[46]
32	PUBMED	Case-control	The levels of 14 cytokines were significantly elevated in COVID-19 cases and showed different expression profiles in patients with different disease severity. Expression levels of IFN-γ-induced protein 10, monocyte chemoattractant protein-3, hepatocyte growth factor, monokine-induced IFN-γ and macrophage inflammatory protein 1 alpha were shown to be associated with disease severity, were notably higher in critically ill patients, followed by critically and moderately ill patients. IFN-γ-induced protein 10 and monocyte chemotactic protein-3 were excellent predictors for COVID-19 progression.	[47]
33	PUBMED	Clinical trial	Initial plasma concentrations of IL-1B, IL-1RA, IL-7, IL-8, IL-9, IL-10, basic FGF, G-CSF, GM-CSF, IFN-γ, IP-10, MCP-1, MIP-1A, MIP-1B, PDGF, TNF-α and VEGF were higher in both ICU and non-ICU patients compared to healthy adults. Additional comparisons between ICU and non-ICU patients showed that plasma concentrations of IL-2, IL-7, IL-10, G-CSF, IP-10, MCP-1, MIP-1A and TNF-α were higher in ICU patients than in non-ICU patients.	[48]
34	PUBMED	*In vitro* assay	Although all three IFN subtypes reduced lung proliferation after treatment during recovery from influenza, only endogenous IFN-λ compromised repair. Patients with COVID-19 exhibited strong induction of IFN and p53 signalling in BALF (from bronchial alveolar lavage fluid) samples collected. Treatment with IFN-λ early in influenza virus infection is protective in mice, offering antiviral protection without the pro-inflammatory responses associated with IFN-α/β. By studying specific effects on the respiratory epithelium, a mechanism was identified whereby excessive or prolonged IFN production exacerbates respiratory viral disease independent of immunomodulation. IFN-induced p53 protein directly reduces epithelial proliferation and differentiation, which increases disease severity and susceptibility to bacterial superinfections.	[49]
35	PUBMED	*In vitro* assay	In mice, IFN-λ produced by lung DCs in response to a synthetic viral RNA has been shown to induce barrier damage, causing susceptibility to lethal bacterial superinfections.	[50]
36	PUBMED	Cohort study	IL-6 was one of the most robust prognostic markers of survival, while elevated TNF-α was a strong predictor of worse prognosis. IL-8 showed an association with survival time, although it was eclipsed by other severity factors after multivariate adjustment.	[51]
37	PUBMED	Cohort study	The immune profile revealed a general increase in innate cell lineages, with a concomitant reduction in the number of T cells. An early elevation in cytokine levels was associated with worse disease outcomes. A ‘principal signature of COVID-19’ was observed that was shared by both moderate and severe disease groups and was defined by the following inflammatory cytokines, which correlated positively with each other: IL-1α, IL-1β, IL-17A, IL-12, p70 and IFN-α. Unsupervised clustering analysis identified four immune signatures, representing growth factors (A), type 2/3 cytokines (B), mixed type 1/2/3 cytokines (C) and chemokines (D) that correlated with three distinct disease trajectories. The immune profiles of patients who recovered from moderate COVID-19 were enriched in tissue repair growth factor signature A, while profiles of those with severe disease developed elevated levels of all four signatures. In patients with severe disease, an additional inflammatory cluster defined by thrombopoietin (TPO), IL-33, IL-16, IL-21, IL-23, IFN-λ, eotaxin and eotaxin 3 was observed. Most cytokines linked to cytokine release syndrome (CSR), such as IL-1α, IL-1β, IL-6, IL-10, IL-18 and TNF, showed increased positive associations in patients with severe disease. Although there was no significant difference in viral RNA load between patients with moderate and severe disease at any specific time point analysed, patients with moderate disease showed a steady decline in viral load over the course of the disease, while those with severe disease did not.	[52]
38	PUBMED	Review	Negative regulation of the human leucocyte antigen DR (HLA-DR) isotype in monocytes appears to correlate with disease severity. Neutrophils are also affected in severe COVID-19, as a result of emergency haematopoiesis generating circulating or developing neutrophil precursors, which have a unique gene expression profile that is similar to that of plasmablasts. NK cells are also activated in COVID-19, and distinct NK cell immunotypes can be associated with disease severity. Also, men tend to have worse COVID-19 results than women, and this may be due in part to immunological differences. For men tend to have more robust production of innate cytokines and non-classical monocytes, while women have more robust T-cell responses.	[53]
39	PUBMED	Cohort study	M-MDSC indices were elevated in the blood but not in nasopharyngeal or endotracheal aspirates of COVID-19 patients compared with healthy controls. M-MDSCs isolated from COVID-19 patients suppressed T-cell proliferation and IFN-γ production partially through an arginase 1-dependent (Arg-1-dependent) mechanism. The patients also showed increased plasma levels of Arg-1 and IL-6.	[54]
40	PUBMED	Cohort study	Type I IFN-dependent TLR3 and IRF7 inborn errors of immunity at eight *loci* were found in up to 23 patients (3.5%) of various ages (17–77 years) and offspring of various nationalities (being from Asia, Europe, Latin America and the Middle East) and in patients of both genders.	[55]
41	PUBMED	Review	The mechanisms of NETs release in the viral response appear to involve the production of neutrophils, toxic factors, viruses and pro-inflammatory cytokines such as TNF-α and IL-8. Overproduction of NETs induces lung tissue damage by NET-related enzymes such as neutrophil elastase (NE) and myeloperoxidase (MPO), also related to local inflammation in the lung and patients with SARS. In addition, dysregulated production of NETs is associated with disease severity and extent of lung injury. The inflammatory process is a gateway to thrombotic complications commonly seen in patients with COVID-19, and immunothrombotic dysregulation appears to be an important marker for disease severity.	[56]
42	PUBMED	Clinical trial	Immunity induced by tuberculosis vaccine, Bacillus Calmette–Guérin (BCG), can affect the susceptibility of different populations to COVID-19 and/or severe disease. BCG in healthy human volunteers results in increased production of pro-inflammatory cytokines, such as IL-1β, TNF and IL-6. In this way, BCG leads to epigenetically trained monocyte and/or NK cell populations, which likely reside in the bone marrow. Upon activation with PAMPs, which can be from bacteria or viruses, these cells show an increased response, promoting host defense.	[57]
43	PUBMED	Case-control	In severe cases, the number of T and B lymphocytes, DCs, NK cells and cells expressing HLA-DR decreased dramatically. In mild cases, the decline in lymphocyte numbers was less pronounced and innate immunity was preserved, as indicated by an increased number of activated myeloid and DCs, as well as NK cells that expressed the activation marker at the same level as controls, and low expression of the M2 marker in the monocyte population.	[58]
44	PUBMED	Case-control	It was reported that the innate immune response of male patients was characterised by elevated plasma levels of cytokines such as IL-8, IL-18, CCL5, chemokines and the induction of these unconventional – ncMono mononuclear cells (CD14- and CD16+) compared to women. Limited T-cell response was clinically associated with further disease progression in male patients. However, higher levels of innate immune cytokines were associated with worsening COVID-19 in female patients. Furthermore, interestingly, IL-6 levels after viral infection were lower in women than in men.	[59]
45	PUBMED	Review	Structurally related TLR7 and TLR8 are encoded on the female X chromosome, so such genes may represent gender-related risk factors. One of the two female sex chromosomes is usually inactivated in females, but the TLR7 – and probably TLR8 – genes escape this silencing, indicating that cytokine induction appears to be dose-dependent on the amount of genetic material detected from the virus. An immune response activated via the TLR7 and/or TLR8 pathway may also induce unwanted side effects. Furthermore, the TLR7 and TLR8 genes exhibit copy number variation (CNV) in the population, thus TLR genes that exhibit allelic polymorphisms may influence the strength of the interaction with their respective ligands or in the quality of the resulting signal that is transduced to the nucleus. Finally, there are indications that gender-based CNV of TLR7 has medical implications. As a result, high TLR7 copy number loading may ensure a better protective response to ssRNA viruses.	[60]
46	PUBMED	Review	ACE2 is encoded on the X chromosome, but studies have shown that ACE2 expression is lower in the lung tissues of women compared to men, and it has been suggested that oestrogen negatively regulates ACE2 expression. Thus, lower expression of ACE2 may have a protective effect. TMPRSS2 expression is increased by androgens and increased expression in male sputum cells has been described. It was reported that pDCs from women produce more IFN-α/β in response to TLR7 ligands, including viral RNAs, resulting in greater induction of ISGs in women. Both X chromosomal factors and sex hormones have been implicated in these sex differences in type I IFN responses.	[61]
47	PUBMED	Review	Depending on the cell type, 17β-oestradiol, present in women, promotes increased numbers of neutrophils and NK cells in response to viral infection, which may reduce cytotoxicity. In addition, 17β-oestradiol has bilateral effects on monocytes and macrophages. At low doses, the hormone stimulates the release of IL-1, IL-6 and TNF-α. However, high concentrations limit the production of inflammatory cytokines. In contrast, the male hormone has an immunosuppressive effect and administration of exogenous testosterone reduces TLR4 in isolated rat macrophages and effectively reduces their propensity for innate immune response activation. ACE2 expression and enzyme activity varied by sex across the lifespan, where in normal ageing is characterised by increased ACE2 expression in males and females. RNA, protein levels and renal ACE2 activity were increased in male rats under basal conditions. In addition, 17β-oestradiol inhibits ACE2 activity after transcription. The effects of ovariectomised rats recovered after administration of exogenous Ang 1–7 or 17β-oestradiol. Gender differences in the response to high-fat diet-induced obesity were found in females produced more oestrogen-dependent ACE2 and Ang1–7 activity despite weight gain, body mass and fat mass than males. In contrast, male animals were more susceptible to obesity-induced hypertension because they showed increased plasma Ang II, decreased Ang 1–7 and decreased renal ACE2 activity. The positive feedback mechanism of negative ACE2 regulation promoted by abnormal activation of RAS and ADAM17 may explain the increased susceptibility of males to COVID-19. ADAM17 provides a strong link between aberrant immune system activation and exacerbation of protective ACE2, which may promote increased susceptibility to severe COVID-19, including in patients with comorbidities. Oestrogen reduces platelet aggregation, the opposite effect of testosterone. The pathogenesis of RAS depends on the activation of AT 1 R by Ang II. Although AT 1 R expression does not follow a gender-specific pattern in a mouse model of vascular injury, women have been shown to be able to regulate the expression of antagonists. The higher the AT 2 R, the more anti-inflammatory and protective it is in cardiovascular diseases.	[10]
48	PUBMED	*In vitro* assay	Ovariectomy in spontaneously hypertensive rats increased AT 1 R expression in mesenteric vessels and decreased renal AT 2 R expression in Wistar-Hanoverian rats. The addition of extrinsic oestradiol reduced AT 1 R and increased the expression of AT 2 R. Testosterone may mediate the opposite effect, but the evidence is uncertain. Female mice are more likely to positively regulate ACE2 than male mice after Ang II infusion-induced hypertension, increase Ang II catabolism, and decrease glomerular expression of AT 1 R.	[62]
49	PUBMED	Review	The mTOR inhibitors (mechanistic target of rapamycin) play their role in the association of MyD88, IRF7 and TLR9 pathways. The primary myeloid differentiation response 88 (MyD88) and the TIR domain-containing adaptor inducing IFN-β) (TRIF, also known as TICAM1) are the two major pathways for TLR signal transduction, which provide the most effective antiviral defense against SARS-CoV-2. TNFR receptor-associated factor family (TRAF) and interleukin-1 receptor-associated kinase (IL-1R) family (IRAK) proteins in the signalling pathways cause activation of NF-κB) and IRF, which in turn leads to the production of type I IFN and pro-inflammatory cytokines such as IL-1, IL-6, TNF-α and IL-12. The pathological role of TLR4 in excessive inflammation in patients with COVID-19 was highlighted, as it leads to NETosis and activation of the inflammasome, which leads to pyroptosis. Moreover, prophylactic administration of TLR2/6 agonist reduces SARS-CoV-2 transmission and provides protection against COVID-19. Stimulation of TLR2 leads to activation of the innate immune response, suppression of excessive inflammation and tissue damage, as well as promotion of the integrity of the local epithelial barrier function.	[63]
50	PUBMED	*In vitro* assay	It was found that, compared to Sendai virus (SeV) infection or poly I:C transfection, SARS-CoV-2 infection induces a delayed increase in IFNs, ISGs and inflammatory cytokines, resulting in limited infection and dissemination in lung epithelial cells. Interestingly, MDA5, RIG-I-like RLR receptor (LGP-2) and NOD1, but not RIG-I, are required for SARS-CoV-2 recognition in lung epithelial cells. In addition, IRF3, IRF5 and RelA (p65) act as the main transcription factors that trigger the induction of IFNs.	[64]
51	PUBMED	Review	The nsp1 protein promotes the degradation of IFN-β mRNA, while ORF6 disrupts IFN induction by preventing the transport of IRF3 and STAT1 into the nucleus. This occurs via two mechanisms, with ORF6 and ORF3b reducing IFNAR signalling by disrupting nuclear import of STAT1 and promoting proteolytic degradation of STAT1, respectively. ORF4a interacts with dsRNA and the RIG-I-like receptor cofactor (PACT), inhibiting IFN induction. ORF4b blocks IFN induction by binding to TBK1 and IKKε (IκB kinase-ε). It was reported that ORF3b is one of the most common antigens recognised by antibodies during the early stage of infection, indicating that ORF3b is highly expressed in the acute stage of infection and may represent an immunodominant epitope. It has been suggested that nsp13, nsp14, nsp15 and ORF6 function as IFN antagonists by suppressing the nuclear localisation of IRF3. SARS-CoV-2 nsp10 interacts with the endocytosis regulator AP-2, a critical regulator of MHC II traffic to antigen-loading compartments. In addition, the M protein of SARS-CoV-2 through interaction with the RIG-I/MDA-5 (melanoma differentiation-associated protein 5)-MAVS signalling pathway inhibits the production of type I and III IFNs.	[65]
52	PUBMED	Case-control	A significant increase in blood IFN-α levels was associated with improvement in the clinical picture in COVID-19. Conversely, a trend was found for IFN-β, where levels of IFN-β below the limit of detectability in a substantial proportion of subjects. Significantly higher blood lymphocyte values and lower IL-10 levels were found in patients who survived. In patients who improved clinically and survived during the study, we found an inverse association between IL-10 and IFN-α levels.	[66]
53	PUBMED	Review	It suggests that the N protein of SARS-CoV-2 is a potent activator of the mannose-binding lectin (MBL)/MBL-associated serine protease (MASP) pathway and may be involved in the rapid development of SARS in SARS-CoV-2-infected patients. Patients with the strongest anti-COVID-19 antibody response may develop more severe ARDS, possibly due to activation of the alternative and mannose lectin binding pathway (MBL/MASP) of complement by Ig-G/SARS-CoV-2 complex and thrombotic events. There is an association of COVID-19-related inflammation with activation of the C5a–C5a receptor (C5aR) axis.	[67]

Figure 2 shows the (A) entry of the virus through the alveolar epithelial cell through the binding of spike protein (S) with ACE2 cleaved by TMPRSS2.

(B) It causes the recognition of APCs through their PRRs, such as, primarily AIM2, NLRs, RLRs, TLRs (TLR2, TLR4 and TLR6) that identify the PAMP of SARS-CoV-2. TLR pathways recruit important downstream adapter proteins, such as TRAFs, MyD88, interferon regulatory factors (IRFs) and nuclear factor-kappa B (NF-κB) for induction of IFN-I (driven by IFNAR1 and IFNAR2), IFN-III.

(C) Inside the endosomes, TLR3, TLR7/8 and TLR9 operate as PRRs recruiting important downstream adapter proteins to recognise the PAMPs from the virus and stimulate the IFN response.

(D) The virus generates immunological dysregulation through its replication and maturation, in which, through the host cell, it produces structural and non-structural proteins. The membrane (M) inhibits the generation of IFN-I and IFN-III. Regarding the non-structural, Nsp proteins play a major role in virulence, such as Nsp1, Nsp3, Nsp12, Nsp13, Nsp14, Nsp15 and Nsp16, which have shown to have a potential role in the inhibition of IFNs. Furthermore, ORFs such as ORF3, ORF3a, ORF3b, ORF4a, ORF4b, ORF6, ORF8 and ORF9b inhibit TLR pathways such as NEMO and IRFs and IFN-signalling pathways.

(E) The envelope (E) induces NLRP3 inflammasome activation that generates the pyroptosis and the nucleocapsid (N) activates the complement lectin pathways (MBL and MASP), which play a pro-inflammatory role in the patient.

(F) The pathogenesis of the virus can cause fibrosis, oedema, thrombotic events, lung damage and a high inflammatory process. Pro-inflammatory cytokines and chemokines, such as interleukin-1 (IL-1), IL-1α, IL-1β, IL-2, IL-1RA, macrophage colony stimulating factor (M-CSF), granular colony stimulating factor (G-CSF), IL-6, IL-10, IL-12, IL-15, IP-10, macrophage inflammatory protein 1-alpha (MIP-1α), chemokine ligand 3 (CCL3), tumour necrosis factor-alpha (TNF-α), IL-8, IL-18, CCL2, IL-2R, IL-33, IL-21 and IL-17 have been associated with disease severity.

(G, H) In severe cases of COVID-19, innate immune cells such as macrophages are increased in mixed polarisation M1/M2 (with FABP4+ phenotype, which is associated with a persistently hyperinflammatory phenotype profile in a hyperactivated state), there is induction of CD14^+^ and CD16^+^ monocytes with lower expression of HLA-DR (defining an acquired immune-suppression), reduction of natural killer (NK) cells (with different immunotypes and higher expression of HLA-E, NKG2A and NKG2C, causing overactivation of NK cells), high recruitment of neutrophils with production of neutrophil extracellular traps (NETs) that create a situation of NETosis in the alveoli, high level of neutrophils in proportion to lymphocytes, reduction of T cells of the mucosa and of total DCs (from plasmacytoid dendritic cells – pDCs and mature dendritic cells – mDCs). Platelets stimulate growth factors such as FCFb and PDGF that promote cellular proliferation, collagen production and other elements of the cellular matrix, enhancing the healing process, even under adverse conditions. pDCs and macrophages show a complete response to SARS-CoV-2, launching a late but robust IFN type I response and releasing other inflammatory cytokines against the virus. Both type I and type III IFNs are induced at high levels in pDCs upon SARS-CoV-2 stimulation.

(I) Innate immunity seems to affect and protect the sexes differently, due to several factors, such as possibly increased production of the activate disintegrin and metalloproteinase (ADAM17) gene in men, reduction in the typical RAS pathway, increased activation of angiotensin II (AngII or Ang1–7), high recruitment of neutrophils, NK cells, monocytes and macrophages and, in women, a lower level of ACE2, which allows greater generation of interferon-stimulating genes (ISGs), with more IFNs, with a lower inflammatory picture of cytokines in females, which despite that, is more effector in males, with production of IL-8, IL-18, CCL5 and unconventional monocytes.

## Discussion

Despite multifaceted efforts, certain treatments for SARS-CoV-2 have shown controversial benefits [68–72]. These include remdesivir, immunoglobulins, monoclonal antibodies to protein S and anti-inflammatory agents. New vaccines are alternatives to stop the progression of the pandemic. However, in the early stages of the outbreak, attention should be focused on treatments designed to control the cytokine storms that occur in patients with SARS-CoV-2 pneumonia. Efforts have been made to control the components of innate immunity that may contribute to the morbidity and mortality of SARS-CoV-2 pneumonia. Thus, understanding the structure of novel viruses and their interactions with the immune system is essential for drug and vaccine production.

According to Mason, the pathogenesis of COVID-19 in 2020 can be divided into three stages: the first stage is asymptomatic and occurs when the inhaled SARS-CoV-2 virus begins to replicate in nasal epithelial cells; the second stage occurs when the virus passes through the lungs, continues to spread, and triggers a stronger innate immune response and the third stage manifests as hypoxia, ground-glass infiltration and development of respiratory failure [73]. Clinical presentations range from no symptoms to mild fever, cough and dyspnoea to cytokine storm, respiratory failure and death. The mechanisms used by CoV to drive IFN responses can be divided into three categories: evasion (the virus protects itself from PRR), as a strategy to drive dsRNA replication (produced as a mediator of transcription) in double-membrane vesicles; inhibition of IFN induction by the virus, which inhibits IFN transcription; inhibition of IFN signalling with the help of its eight specific viral proteins that suppress IFNAR signalling (IFNα/β receptor) [65].

SARS-CoV-2 appears to act on the activation and maturation of IL-1β, which in turn activates other pro-inflammatory cytokines, such as IL-6 and TNF-α. The immunopathology of COVID-19 is characterised by an elevation of IL-6 and TNF-α. These cytokines are products of activation of the TLR4, which is part of innate immunity. IL-1 levels are related to the virulence and severity of the infectious process [74].

IL-6 contributes to the host's defense against infections and tissue injury. However, exaggerated and excessive synthesis of IL-6 accounts for its elevated serum levels in COVID-19 patients and is associated with disease severity due to its ability to recruit different cell immunotypes in innate immune response. Patients infected with COVID-19 harbour an expanded population of circulating monocytes that secrete IL-6 and IL-1β, as a result, patients with COVID-19 have elevated serum IL-6 as well as lactate dehydrogenase levels compared with healthy controls [75]. TNF-α is generated by macrophages, monocytes, endothelial cells, neutrophils, smooth muscle cells, activated lymphocytes, astrocytes and adipocytes. It is associated with the disease severity by sustaining inflammatory milieu and plays a role in the formation of oedema [76].

*In vitro* stimulation by IL-6 and soluble IL-6 receptor previously revealed impaired cytolytic functions (perforin and granzyme B production) by NK cells from healthy donors, which can be restored after addition of tocilizumab (IL-6R blockade) [48]. Although it is the main cause of cytokine storms in COVID-19 patients, IL-6 has both anti-inflammatory and pro-inflammatory properties that play a complex role in COVID-19 pathology.

Upon virus entry, SARS-CoV and SARS-CoV-2 activate disintegrin and metalloproteinase (ADAM17), which are cellular events that help differentiate mild and severe coronavirus infections. Importantly, activation of the standard RAS pathway (ACE/AT 1R) resulted in an increase in ADAM17 activity. Therefore, hyperactive acute respiratory distress syndrome (ARDS) promotes disease severity, especially in patients with cardiovascular diseases as comorbidities [10].

Innate immune cells appear to be responsible for the inflammatory environment in the lungs. Cytokine storms are a complex network of interactions between immunologically important molecules and molecular events expressed in the clinical phenotype of systemic inflammation, multiple organ failure and increased blood potassium. This framework releases large amounts of pro-inflammatory cytokines, as in COVID-19, including IL-6, TNF, IL-1β, IL-12, IL-17, IL-18, IP-10, IFN-γ, CCL2 and CCL5 [43, 77]. In addition to further stimulating the cytokine storm, SARS-CoV-2 was able to infect DCs and limit DC maturation, thus suppressing T-cell responses. Three studies demonstrated a high proportion of CD14^+^ and CD16^+^ cells in patients with severe COVID-19 [46, 78, 79], with inflammatory factors including GM-CSF and IL-6, IL-10 and TNF-α. Alveolar FABP4+ macrophages predominate in severely damaged lungs. The alveolar FABP4+ macrophage induces viral inflammation because it produces high levels of IFN and chemokine-stimulated genes [21, 80]. These results suggest an association between elevated levels of GM-CSF and IL-6 secreted by monocytes and cytokine storm.

The IFN-mediated antiviral pathway is one of the primary mechanisms of innate immunity against invading viral pathogens and consists of two steps, including IFN activation and IFN signalling. First, viral infection rapidly induces the expression and secretion of type I and type III IFNs. In the terminal phase, secreted type I and type III IFNs bind to cell surface receptors and initiate Janus kinase (JAK) and transcriptional activator (STAT) signalling. When IFN binds to these receptors, intracellular tyrosine kinases, including JAK1, JAK2, JAK3 and tyrosine kinase 2 (TYK2), are activated and phosphorylated, resulting in increased phosphorylation and activation of STAT1 and STAT2 [40].

INF type I is considered the first line of defense against viruses [41]. IFN-I is a family of cytokines that bind to the IFN receptor and is composed of two transmembrane subunits, IFNAR1 and IFNAR2. The two receptors include an extracellular domain that binds to IFN-I, a transmembrane helix and an unstructured intracellular domain that binds to JAK and STAT (signal transducers and transcription activator signalling pathways) transcriptional activator signals. Type I IFN via IFNAR activates JAK and STAT. After IFNAR signalling, JAK1 and TYK2 phosphorylate STAT1 and STAT2 molecules to form a complex with IRF9 and interferon-stimulating gene factor 3 (ISGF3). Type I IFNs correspond to IFN-α, IFN-β, IFN-ε, IFN-ω and IFN-κ and the IFN-II and IFN-III families include IFN-γ and IFN-1. Consequently, IFN-α strongly inhibits SARS-CoV-2 replication *in vitro* [81]. In the case of IFN-β, after ORF6 binds to its receptor it activates the JAK-STAT pathway, in which the kinases JAK1 and TYK2 phosphorylate STAT1 and STAT2, triggering their dimerisation and nuclear translocation [19].

These complexes are inserted into the nucleus and stimulate the transcription of ISGs, followed by the expression of antiviral proteins. IFN-stimulating genes are an integral part of the innate antiviral defense mechanism that limits viral invasion and restricts viral replication after the virus invades the host cell. Several ISG products, including IFN-inducible transmembrane proteins (IFITM) 1, 2 and 3, limit SARS-CoV-2-mediated infection [82]. From an immunological point of view, IFN-I has three main functions: to activate the antiviral state of infection and adjacent cells, limiting the spread of infection; to regulate innate immune responses, such as antigen presentation and NK cell function, limiting the inflammatory pathway; to activate the adaptive immune system to develop specific T and B cell responses to high-affinity antigens [83].

Class III IFN (IFN-λ) showed stronger antiviral function than IFN-α in treating influenza infection without activating IFN-α-induced inflammation and tissue damage. On the other hand, IFN-λ treatment has been shown to interfere with the detection of bacteria by lung neutrophils during superinfection with influenza, which alters the lower respiratory tract, weakens defenses and the risk of coinfection of patients with COVID-19 may increase. Similar to IFN-I, IFN-λ is reduced during infection with COVID-19, and IFN-λ suppresses SARS-CoV-2 replication *in vitro* in human enterocytes [18, 49, 50, 65].

Defective type I IFN immunity was shown to underlie life-threatening COVID-19 pneumonia. Genetic defects observed in critically ill COVID-19 patients are acquired during disease evolution through secondary events, in deficient pDCs. Despite this, the specific role of DCs in the COVID-19 pathology has been insufficiently studied, so far [55, 84].

Thus, with reduced serum levels of DCs and plasmacytoid DCs, the combined effects of a naturally poor IFN-inducing viral strain and intrinsic defects in antiviral immune or epithelial responses within the nasal mucosa may predispose to severe disease. These occur due to increased viral replication in the upper airways. NK cells have the role of recruiting their different immunotypes to exert their cytotoxicity. Furthermore, NK cells exhibit an activated and cyclic phenotype in acute SARS-CoV-2 infection at both the protein and transcriptomic levels, with upregulation of Ki67, CD69, HLA-DR and CD38. The upregulation of inhibitory checkpoint receptors such as LAG3, TIGIT and TIM3 reduces the formation of NK cells [22, 24].

The N protein catalytically modifies the host protein ACE2 by SUMOylation and ubiquitination, respectively, by interaction with the host proteins hUbc9 and TRIM25, respectively, as well as acting as an antagonist of IFN-I production. The circadian shift of ubiquitination towards the N protein as a RIG-I target for proteolysis, thereby redirecting cells away from critical virus detection PRRs is well described, while the target of N-mediated SUMOylation remains unclear. Elevated levels of IL-6, IL-10, TNF-α and interleukin 2 receptor (IL-2R) are associated with disease severity. In addition to manipulating cytokines, CoV also controls other immune processes, including antigen presentation [40, 85].

It has been hypothesised that the hyperinflammatory innate immune system caused by SARS-CoV-2 infection cannot be eliminated due to its antagonism of the innate immune response [32]. Therefore, it induces an excessive release of inflammatory cytokines and compensates for the depletion of the immune system caused by SARS-CoV-2-induced lymphopaenia [86]. In addition, TLR3- and IRF7-dependent IFN-I innate errors of immunity have been found and related to disease severity. The most thought-provoking are the autosomal-recessive (AR) deficiencies of IRF7 and IFNAR1. The AR form of IFNAR1 deficiency highlights the importance of type I IFN production over type III IFN production, which is also impaired by defects of TLR3, IRF7 and IRF9 [55].

Regarding the pulmonary aetiology, the overproduction of cytokines after SARS-CoV-2 infection increases the permeability of the capillary wall membrane around the infected alveoli, causing pulmonary oedema, dyspnoea and hypoxaemia [87]. Plasma exudation and loss of alveolar elasticity due to reduced surfactant production owing to SARS-CoV-2 infection of type 2 lung cells induces ARDS in patients with COVID-19. Following cytokine storms, these SARS-CoV-2-induced immune disturbances can lead to increased microbial infections, septic shock and severe multiple organ failure [47].

Another important pathological consideration in patients with SARS-CoV-2 pneumonia is their propensity for thrombotic events. Alternative and lectin pathways are effector mechanisms of innate immunity. Many symptoms can result from activation of the complement system by alternative and likely lectin binding pathways. This includes the tendency to ARDS and the potential for hypercoagulability. Strong complement activation can induce activation of the coagulation system (with the appearance of thrombotic events in endothelial vessels). Therefore, inhibition of complement activation may prevent thrombotic complications of SARS-CoV-2 pneumonia [67].

TLR agonists can also be used as prophylaxis for SARS-CoV-2. Proud *et al*. [88] demonstrated that prophylactic use of a TLR2/6 agonist reduces SARS-CoV-2 infection and protects against COVID-19. TLR2 stimulation leads to activation of the innate immune response, suppression of excessive inflammation and tissue damage, as well as generating the integrity of local epithelial barrier function. TLR promotes the recruitment of Toll-interleukin (TIR) domain-containing adaptor proteins, such as myeloid differentiation factor 88 (MyD88), TIR domain-containing adaptor protein (TIRAP) and TIR domain-containing adaptor protein inducing IFN-β-related adaptor molecule (TRIF), leading to activation of the transcription factors IFN regulatory factor-3 (IRF3), IRF7 and nuclear factor kappa enhancer light chain B activated cells (NF-κB) required for the transcriptional induction of antiviral IFN, pro-inflammatory cytokines and chemokines [21, 88].

In particular, activation of the IL-6 pathway by SARS-CoV-2 infection can induce vascular endothelial growth factor (VEGF), fibrinogen and factor VII. IL-6 blockade or reduced induction may be a new treatment strategy for critically ill patients with COVID-19 [89, 90]. Ang II also stimulates the expression of TFs (tumour inflammatory factor mediators) in affected cells, increases thrombin formation and impairs fibrinolysis [91].

Other anti-inflammatory agents were also tested in their effectiveness by repurposed drugs for COVID-19 therapy and involved positive results under mediators such as inhibitors of C5, IL-1, JAK1, JAK2 and IL-33 and related to reducing release of TNF-α and IL-1β [92].

Since thrombosis is one of the physiologically related events caused by continuous activation of the Ang II signalling pathway, ACE2 deficiency was stimulated by the pro-inflammatory condition caused by SARS-CoV-2 and may exacerbate thrombosis. It is unclear whether IFN-I therapy is an effective benefit for patients with COVID-19, since SARS-CoV-2 may use ISG to increase infectivity, and it is not known whether the IFN response limits SARS-CoV-2 replication. However, some clinical studies have shown positive data on IFN-I as a form of treatment or prevention of COVID-19 [93–97].

IFN levels and duration in a variety of human diseases can be associated with determining macrophage/monocyte resistance. Induction or suppression of tolerance is mediated by transcriptional, epigenetic and metabolic reprogramming [98], which leads to dysregulation of the inflammatory response. Therefore, dramatic epigenetic and metabolic changes, possibly associated with macrophage resistance and innate immune memory have recently been reported in patients with COVID-19 [21].

Disruption of the immune system is one of the consequences of COVID-19. However, there are differences in the immune system between mild and severe conditions. In mild cases, the innate immunity seems to be maintained. On the other hand, in severe cases, the innate systemic process is affected. Congenital lymphoid cells (ILCs containing NK cells) have no specific antigen receptors, but are sometimes highly reactive to specific pathogens or are referred to as ‘trained immunity’ [99].

The number of NK cells and DCs (total and activated) decreased in severe cases. The number of cells expressing HLA-DR is significantly lower in severe cases, indicating that antigen-presenting cells are unable to induce an adaptive response. The virus also affects the monocyte population. The proportion of intermediate and unconventional monocytes has decreased, indicating a decrease in functional maturity. An increased proportion of cells co-expressing M1 and M2 monocyte markers suggests the development of fibrosis as a mechanism of prolonging and repairing inflammation, potentially damaging the lung parenchyma and increasing the risk of worsening clinical outcomes [58].

Gender differences in COVID-19 are reflected in the initial treatment of viral infections, various hormonal signalling pathways and risk status based on social and cultural factors. The differences in immune functions between women and men, which are manifested not only by stronger immune responses in women against pathogens and vaccines, but also by greater susceptibility to autoimmune diseases. Many viruses are affected by oestrogen at the molecular level, especially in the mechanism of virion replication and maturation, but so far there is no evidence for SARS-CoV-2 [10].

The literature suggests that the ability of women to induce an early innate immune response is increased and the typical trend towards increased RAS activity in diseases that are strongly dependent on both genetics and sex hormones is reduced. Thus, these candidate pathways and molecular mechanisms provide a multifaceted account of the severity and mortality of COVID-19 in men compared to women. Positive regulation of the ACE2 axis, Ang 1–7 MasR or ADAM17-mediated blockade of ACE2 release, provides an altered pathway essential for ameliorating the increased severity in men [10, 59–62].

Furthermore, defects in the innate immune cells response to SARS-CoV-2 caused by ageing are related to poorly managed viral multiplication and inflammation during the early symptom phase and in the following illness development. These defects in the effect of age (which affects older people more compared to adults and children) are related to reduced activation of monocytes (as a consequence of downregulation of antigen presentation molecules, e.g. HLA-DR) that act as APCs and antiviral mediators, DC dysfunction (that should regulate the initial response against the pathogen) and reduced percentage of cytokine-producing NK cells (as a result of decreased expression of functional immune co-stimulatory molecules). Hence, an underlying pathophysiological reason for the lack of virological control and higher risk of disease progression in older COVID-19 patients is impaired innate cellular responses during the early stages of infection [100].

## Conclusions

To date, SARS-CoV-2 remains prevalent as a major global health emergency due to its pathogenesis. Its immunopathogenesis reveals several immune evasion factors, which are crucial for replication, maturation and invasion into host cells that are determinants for the success of viral activity. Innate immunity appears as indispensable in the initial stage to provide the influx of combined innate immune cells, IFNs (mainly types I and III), cytokines and chemokines, and complement that regulate the longest survival of patients in pictures differentiated from mild, moderate to severe. Thus, the severity of the disease goes through innate errors, virulence of the virus and gender differences, which also correlate with comorbidities. Moreover, it is believed that much remains to be revealed about the real impact of innate immunity and the clarification of controversial theories of both prognosis and therapy on the means of this immunity.

## Data Availability

Data availability is not applicable to this article as no new data were created for this study.
